# *Nyssorhynchus darlingi* genome-wide studies related to microgeographic dispersion and blood-seeking behavior

**DOI:** 10.1186/s13071-022-05219-5

**Published:** 2022-03-28

**Authors:** Marcus Vinicius Niz Alvarez, Diego Peres Alonso, Samir Moura Kadri, Paulo Rufalco-Moutinho, Isabella Ariadne Ferrari Bernardes, Ana Carolina Florindo de Mello, Ana Carolina Souto, Gabriel Carrasco-Escobar, Marta Moreno, Dionicia Gamboa, Joseph M. Vinetz, Jan E. Conn, Paulo E. M. Ribolla

**Affiliations:** 1 Sao Paulo State University (UNESP), Botucatu, 18618-689 Brazil; 2 Nucleo de Medicina Tropical, Universidade de Brasília, Brasília, Brazil; 3 Laboratorio ICEMR-Amazonia, Laboratorios de Investigación Y Desarrollo, Facultad de Ciencias Y Filosofia, Universidad Peruana Cayetano Heredia, Lima, Peru; 4 Facultad de Salud Pública, Universidad Peruana Cayetano Heredia, Lima, Peru; 5 Department of Immunology and Infection, London School of Hygiene and Tropical Medicine, London, UK; 6 Departamento de Ciencias Celulares Y Moleculares, Facultad de Ciencias Y Filosofía, Universidad Peruana Cayetano Heredia, Lima, Peru; 7 Instituto de Medicina Tropical Alexander Von Humboldt, Universidad Peruana Cayetano Heredia, Lima, Peru; 8 Section of Infectious Diseases, Department of Internal Medicine, Yale School of Medicine, New Haven, CT USA; 9 Wadsworth Center, New York State Department of Health, Albany, NY USA; 10 Department of Biomedical Sciences, School of Public Health, State University of New York at Albany, Albany, NY USA

## Abstract

**Background:**

In Brazil, malaria is concentrated in the Amazon Basin, where more than 99% of the annual cases are reported. The main goal of this study was to investigate the population structure and genetic association of the biting behavior of *Nyssorhynchus* (also known as* Anopheles*)* darlingi*, the major malaria vector in the Amazon region of Brazil, using low-coverage genomic sequencing data.

**Methods:**

Samples were collected in the municipality of Mâncio Lima, Acre state, Brazil between 2016 and 2017. Different approaches using genotype imputation and no gene imputation for data treatment and low-coverage sequencing genotyping were performed. After the samples were genotyped, population stratification analysis was performed.

**Results:**

Weak but statistically significant stratification signatures were identified between subpopulations separated by distances of approximately 2–3 km. Genome-wide association studies (GWAS) were performed to compare indoor/outdoor biting behavior and blood-seeking at dusk/dawn. A statistically significant association was observed between biting behavior and single nucleotide polymorphism (SNP) markers adjacent to the gene associated with cytochrome P450 (CYP) 4H14, which is associated with insecticide resistance. A statistically significant association between blood-seeking periodicity and SNP markers adjacent to genes associated with the circadian cycle was also observed.

**Conclusion:**

The data presented here suggest that low-coverage whole-genome sequencing with adequate processing is a powerful tool to genetically characterize vector populations at a microgeographic scale in malaria transmission areas, as well as for use in GWAS. Female mosquitoes entering houses to take a blood meal may be related to a specific CYP4H14 allele, and female timing of blood-seeking is related to circadian rhythm genes.

**Graphical Abstract:**

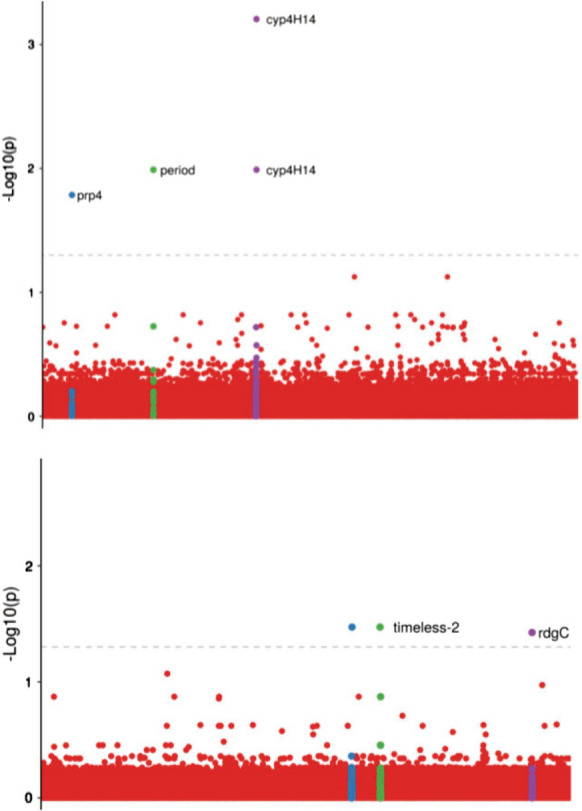

**Supplementary Information:**

The online version contains supplementary material available at 10.1186/s13071-022-05219-5.

## Background

Malaria is the most impactful arthropod-borne disease in developing countries. According to the WHO world malaria report 2019 [1], there were 229 million malaria cases and an estimated 409,000 deaths related to malaria in 2018, 94% of which were concentrated in Africa. In addition to Africa, this disease affects other poor populations in tropical and subtropical areas because environmental conditions are favorable for the development of the vectors and dissemination of the causative agent [2].

Brazil presents a high incidence of malaria, with the majority of the 194,000 cases registered in 2018 concentrated in the Brazilian Amazon rainforest [3]. *Nyssorhynchus darlingi*, the main malaria vector in Brazil, is highly susceptible to human *Plasmodium* and capable of transmitting the parasite inside and outside houses, even when at low density [4]. Preferred breeding sites for this species are collections of clear, shallow water that are shaded, with vegetation and a low salt concentration [5–7]. *Nyssorhynchus darlingi* is both anthropophilic and opportunistic [8, 9] and, as the natural environment becomes more modified, or deforested, local populations tend to cohabit with humans, invading their homes, thereby increasing the importance of this species as a vector [10]. In the Amazon rainforest, it is the anopheline vector that most quickly and efficiently benefits from the changes humans produce to the natural environment [10, 11].

Recent studies support the hypothesis of a *Ny. darlingi* species complex, and the mosquitoes present in the Amazon correspond to one of three lineages within this complex [12]. Moreover, microgeographic scale studies with markers across the *Ny. darlingi* genome have demonstrated genetic differentiation that could represent phenotypic differences related to malaria transmission dynamics [13, 14].

The rapid development of technologies involved in whole-genome sequencing (WGS) has resulted in dramatic reductions in the per base sequencing cost. However, studies that require the sequencing of large numbers of samples remain costly, possibly prohibitively so in some laboratories. One low-cost strategy is genotyping-by-sequencing for low-coverage WGS (L-WGS), which is associated with imputation that provides sufficient genomic information to select markers accurately [15]. The accuracy of variant detection is low in genomes with low coverage depth and tends to have a high false positive rate, but this is attenuated when information between samples is combined, providing good common variant identification power [15, 16]. The inference of genotypes by imputation for both panel-based genotyping and sequencing genotyping has been shown to be accurate, allowing for the potential use of extreme low-coverage WGS (EXL-WGS) to discover variants at a dramatic reduction in cost when compared to standard WGS [17, 18].

Li and collaborators [19] demonstrated that rare variants in L-WGS samples are more challenging to detect because of the difficulty in distinguishing genuine rare alleles from sequencing errors. The number of variants identified is higher when the proportion of polymorphisms among the sequenced individuals in the segregated population is higher. Since different approaches can be conducted in EXL-WGS analysis, the sensitivity of each method must be carefully adjusted as the reduction in coverage inevitably amplifies the possibility of false positive detection.

In the present study, we used low-coverage sequencing markers to investigate the population of *Ny*. *darlingi* collected in Mâncio Lima, Acre state, Brazil. Genetic data were correlated with information such as specimen collection and location (larva or adult), adult capture time, house-to-house capture location (intra- or peridomestic) and distance of larvae capture sites from forest areas.

## Methods

### Sample collection

**Fig. 1:**
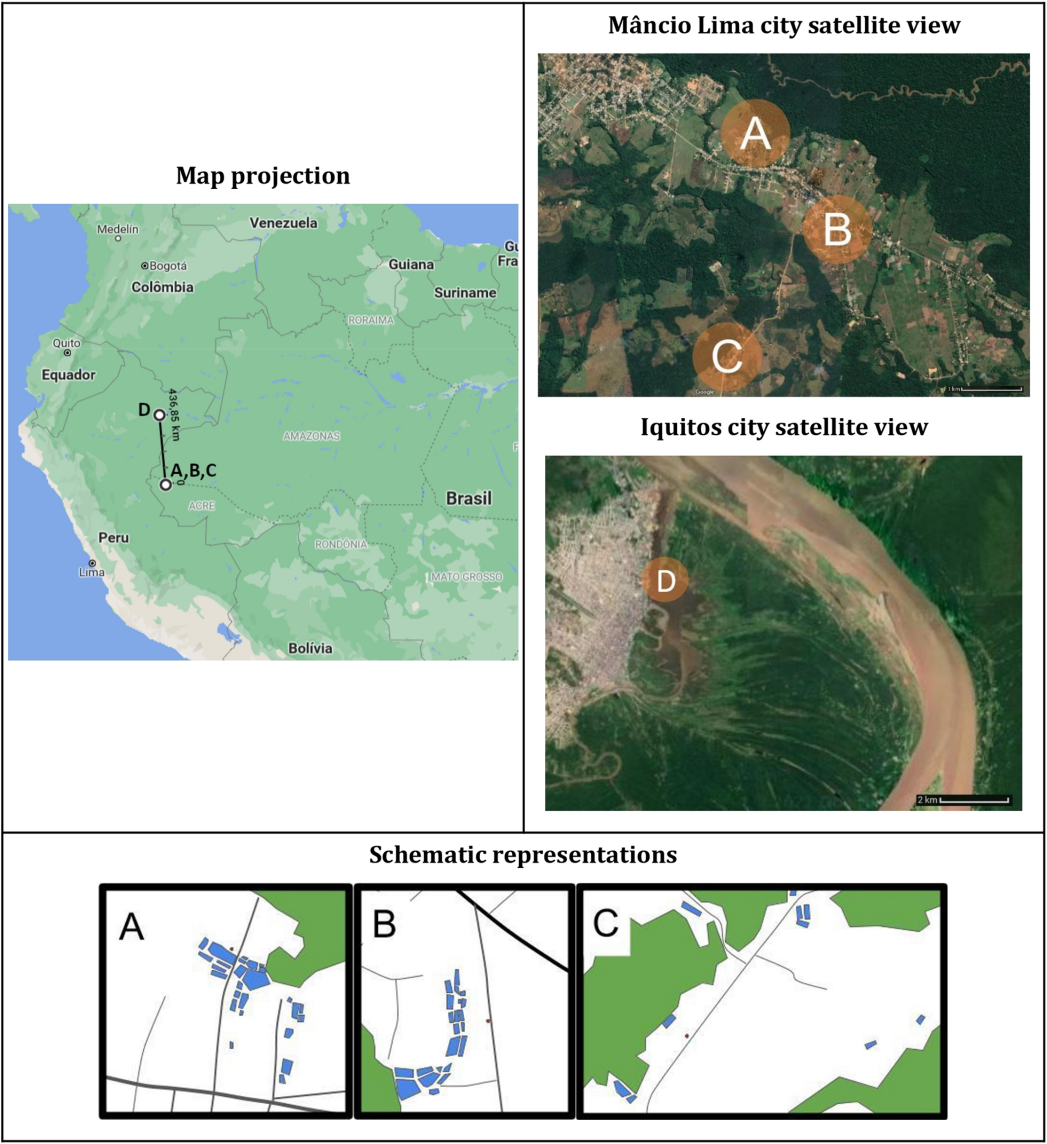
*Nyssorhynchus darlingi* collection sites in Mâncio, Lima city, Acre State, Brazil and in the Salvador community on the Napo River, Iquitos city, Loreto, Peru.* A*–*D* represent the collection sites:* A* 7°37′12.9″S, 72°53′06.7″W;* B* 7°38′02.1"S, 72°52′26.5"W;* C* 7°39′05.3″S, 72°53′20.9″W;* D* 3°44′17.1″S 73°14′19.9″W. The schematic representation of respective sites shows the houses where adults were captured (red dots), all breeding sites analyzed within approximately 1 km of each house (blue), forest areas (green) and the main roads (black lines)

Larval and adult samples were captured from three different collection points in the municipality of Mâncio Lima, Acre state (Fig. 1) during December 2016 and February, May and September 2017. Adult anophelines were collected by human landing catch, by authors DPA, SMK, PRM and PEMR during 12-h collections, from 18:00 to 06:00 hours, for 2 days at each collection point. There were two volunteers indoors and two outdoors, who rotated locations to mitigate collector-specific bias. The three collection points were located around three houses, as depicted in Fig. 1. The three samples are: (i) sampling site A, houses relatively distant from the city center and main streets as well as from forested areas; (ii) sampling site B, houses located in close proximity to the city center, alongside paved streets and distant from forested areas; and (iii) sampling site C, houses relatively distant from city center and in close proximity to forested areas. Biting behavior was recorded and classified as indoor or peridomestic (outdoor). The approximate linear distances between the collection points were: 1.96 km from A to B, 3.39 km from A to C and 2.51 km from B to C. Larvae were also collected in the community of Salvador, Loreto, Peru (sampling site D on Fig. 1), for long-range comparisons (site D is approximately 432 km distant from site A).

### Sample preparation and sequencing

For DNA extraction, heads and thoraces of mosquitoes were separated from the rest of the body with a sterile scalpel. Each adult (head and thorax) and larva (whole body) were extracted individually using the Glass Fiber Plate DNA Extraction Kit (Canadian Center for DNA Barcoding, Guelph, ON, Canada) following the Center’s recommendations. DNA quantification was performed by fluorometric quantitation using QuBit dsDNA HS Assay Kit (Thermo Fisher Scientific, Waltham, MA, USA) according to the manufacturer's recommendations.

DNA libraries were prepared using one fifth of the total volume recommended for the Nextera XT Library prep kit (Illumina Inc., San Diego, CA, USA) following the manufacturer's recommendations. DNA samples were multiplexed to a total of 60 samples per run and sequenced in the NextSeq500 (Illumina) platform in a 151-cycle single-read run. Sequence quality analysis was performed using the FastQC [20] program, and reads were used if results from all analysis modules were approved without errors.

### Species identification

Sequencing data was aligned with the *Ny. darlingi* cytochrome oxidase subunit I (*COI*) reference sequence (available at https://www.ncbi.nlm.nih.gov/nuccore/KP193458.1/) using Burrows-Wheeler Aligner (BWA) software [21]. After alignment, the individual *COI* consensus sequences were generated using the SamTools [22] software package. The BLASTn tool [23] was used for multi-species identification using the individual generated *COI* consensus sequence. Only the highest matching result from BLAST was used. Specimens were discarded if e-value > 1e-100, identities < 200, and identity < 90%, and if the matching sequence was not identified as *Ny. darlingi*.

### Variant calling and genotype imputation

The *Ny. darlingi* reference genome available in the VectorBase database, version AdarC3 [24], was used. Alignments were performed with BWA software and the variant calling was performed with the SamTools software package. A genotype panel was generated in VCF 4.2 format. Single nucleotide polymorphisms (SNPs) were removed from the pre-imputation panel by minimum allele frequency (MAF) < 0.1, as were missing data (MD) > 0.5 using the LCVCFtools program [25]. Genotypes with sequencing depth (DP) < 5 or genotype quality of phred quality score (GQ) < 20 were imputed with BEAGLE 4.1 software [26] using genotype normalized probability values (PL). After imputation, genotypes were removed from the panel if the probability of the imputed genotype (GP) < 0.95. Finally, SNPs were filtered by MAF > 0.1, MD < 0.3 and Hardy–Weinberg Equilibrium (HWE) < 0.001. HWE was calculated within locations (collection points) to test for existing Wahlund effect between groups.

The unimputed genotypic data used in secondary analyses were generated by the same variant call workflow described above, except for the imputation and post-imputation steps. VCF quality control was applied with LCVCFtools. Genotypes were removed if DP < 5 and GQ < 20 and SNPs were filtered for MAF > 0.1 and MD < 0.8 (at least 15 non-missing genotypes from each strata). HWE analysis control was also applied within strata (HWE < 0.001), including samples from Peru.

### Marker selection

The linkage disequilibrium decay was estimated from the *r*^2^ pairwise linkage disequilibrium for all markers, calculated by the PLINK 1.9 program [27]. Linkage disequilibrium averages (*r*^2^) were calculated by 500-bp windows for 40 adjacent windows, for a total of 20 kb. The prediction of the linkage disequilibrium decay function $\hat{Y}$ was calculated according to the nonlinear model $\hat{Y} = \beta_{0} + \beta_{1} \frac{1}{Log\left( x \right)} + e_{res}$ where $\beta_{0}$, is the intercept value, $\beta_{1}$ is the coefficient for variable one over the logarithm of the distance of the markers in base pairs and *e*_*res*_ is the value of residual error. SNPs were selected by pruning, considering as window size that the average *r*^*2*^ value at that distance is approximately between 0.1 and 0.05.

### Population stratification analysis

Stratification signals were estimated by F_*ST*_ according to the mathematical model of Weir and Cockerham [28]. The F_*ST*_ value was calculated using the PLINK 1.9 program according to the model $F_{ST} = \frac{GS - GT}{{1 - GT}}$., where GT and GS are the probabilities that two randomly selected alleles in the population and between house-to-house groups, respectively, will be identical by state. Pairwise F_*ST*_ values were calculated using all pruned markers, and a permutation test (10,000 permutations) was performed to verify the statistical significance of the genome-wide average F_*ST*_ and per SNP F_*ST*_ values. The genome-wide average F_*ST*_ and per SNP F_*ST*_ estimates were considered significant when *P* ≤ 0.05 after false discovery rate (FDR; Benjamini–Hochberg procedure) correction. Principal component analysis (PCA) was performed using the PLINK 1.9 program [27] and k-means clustering was performed using R (R Foundation for Statistical Computing, Vienna, Austria). Both analyses were performed using only the statistically significant SNP for stratification.

### Genome-wide association study

A genome-wide association study (GWAS) was performed using the Cochran-Mantel–Haenszel Test statistical model [29]. The test assumes a case control 2 × 2 × K for *k* strata under the null hypothesis $H_{0}$ that *MH* ~ *χ*^2^ (Chi-square) with 1 degree of freedom. The MH value can be calculated as $\chi_{MH}^{2} = \frac{{\left( {\left| {\mathop \sum \nolimits_{i = 1}^{k} \left[ {a_{i} - \frac{{\left( {a_{i} + b_{i} } \right)\left( {a_{i} + c_{i} } \right)}}{{n_{i} }}} \right]} \right| - \frac{1}{2} } \right)^{2} }}{{\mathop \sum \nolimits_{i = 1}^{k} \frac{{(a_{i} + b_{i} )\left( {a_{i} + c_{i} } \right)\left( {b_{i} + d_{i} } \right)\left( {c_{i} + d_{i} } \right)}}{{\left( {n_{i}^{3} - n_{i}^{2} } \right)}}}}$, given that in any biallelic site (alleles A and B) for the *k*^th^ stratum, *a* and *c* are equal to the total number of alleles A for the case and control, respectively. In the same way, *b* and *d* are equal to the total number of alleles B for the case and control, respectively. *n* is the total number of observed alleles for the *k*th stratum, where *n* = *a* + *b* + *c* + *d*.

Case and control categories were considered indoor and outdoor for biting behavior, and samples were collected between 06:00 and 22:00 hours (dusk); samples collected between 02:00 and 06:00 hours (dawn) were considered as blood seeking at dusk or dawn. Stratum groups were determined by collection location (A, B and C). Table 1 shows the number of samples collected within the studied categories.

The FDR multiple test correction method was applied to control for false positives, assuming statistical significance when corrected *P*-value < 0.05. Manhattan Plot images were generated by R scripting in RStudio [30, 31]. Adjacent genes up to 10 kb from FDR-significant SNPs were investigated using AdarC3 [30, 31] from the annotated *Ny. darlingi* genome available in the gff3 format in VectorBase.

## Results

A total of 436 samples were captured and sequenced, of which 394 and 42 samples were from Mâncio Lima and Salvador, respectively. Following species identification and genome alignment, 73 samples from Mâncio Lima and three from Salvador were discarded due to minimum sequencing coverage threshold, low confidence BLAST result or non-*darlingi* BLAST result for species identification. The samples used in the population analysis are described in Table 1 and Additional file 1: Table A.

**Table 1:** *Nyssorhynchus darlingi *samples identified with BLASTn and *COI* (*e*-value < 1e-100)

Stage	Collection points^a^	Location^b^	Count
Adult	A	Indoor	12
		Outdoor	35
Adult	B	Indoor	7
		Outdoor	15
Adult	C	Indoor	40
		Outdoor	93
Larvae	A	BS 1	9
		BS 2	14
		BS 3	10
		BS 4	15
Larvae	B	BS 1	4
		BS 2	10
		BS 3	11
		BS 4	13
Larvae	C	BS 1	7
		BS 2	13
		BS 3	8
		BS 4	5
Larvae	D	BS 1	39

^a^Mâncio Lima samples were collected at collection sites A (7°37'12.9"S, 72°53'06.7"W), B (7°38'02.1"S, 72°52'26.5"W) and C (7°39'05.3"S, 72°53'20.9"W). Peruvian larvae samples were collected at collection site D (3°23'47.0"S, 73°12'18.2"W)

^b^Adult females were collected indoor or outdoor on each collection point. Larvae were collected around four different breeding sites (BS; 1–4)) within and around each collection point

**Fig. 2:**
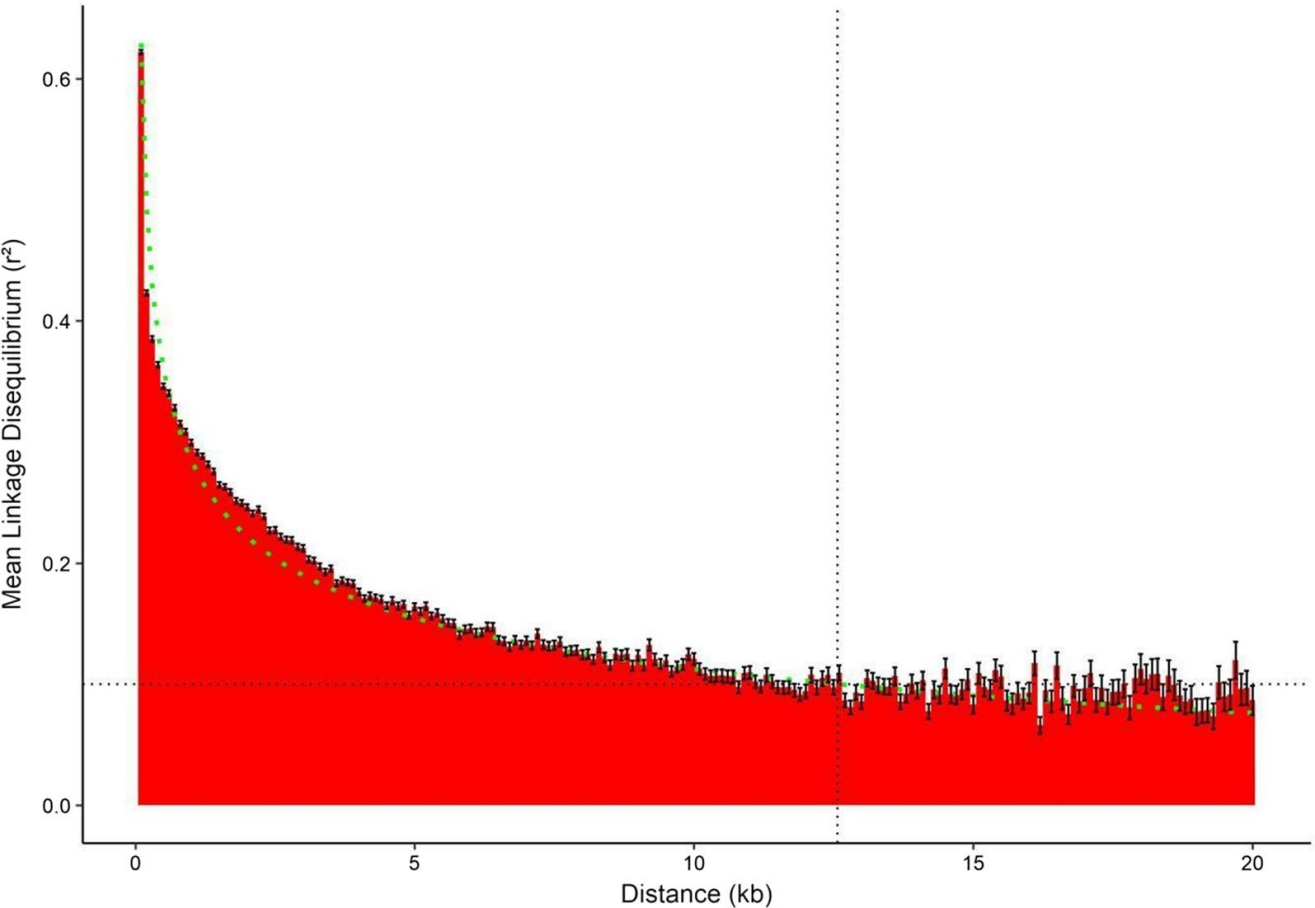
Estimated LD decay. Black dashed vertical line represents the estimated distance (in kbp) for *r*^2^ ≤ 0.1. Black dashed horizontal line represents mean *r*^2^ = 0.1. Black error bars represent the mean standard error. Green dashed line describes the estimated LD decay (nonlinear regression).* Abbreviations*: LD linkage disequilibrium

The imputed genotypes panel from 321 Brazilian samples (Table 1) resulted in 1,070,802 markers, about 8.16 SNPs/kbp and a genotyping rate of 83.6%. The non-imputed genotype panel from 360 Brazilian and Peruvian samples resulted in 330,885 markers (29.6% of the imputed panel), around 2.41 SNPs/Kbp and a genotyping rate of 14.2%. The linkage disequilibrium decay was estimated and the observed nonlinear function coefficients were approximately − 0.40 (*P* < 0.001) and 4.76 (*P* < 0.001) for ${\beta }_{0}$ and ${\beta }_{1}$ , respectively, with *R*^*2*^ approximately = 0.97. At approximately 12.57 kbp away, the estimated average linkage disequilibrium was 0.1 for the lower confidence interval curve. For practical purposes, 14 kb was adopted as the window size for the pruning process. Figure 2 shows the observed average linkage disequilibrium values as a function of distance. Marker selection was performed by pruning, resulting in 123,620 markers (about 0.91 SNPs/kbp) and a genotyping rate of 86.06%.

**Fig. 3:**
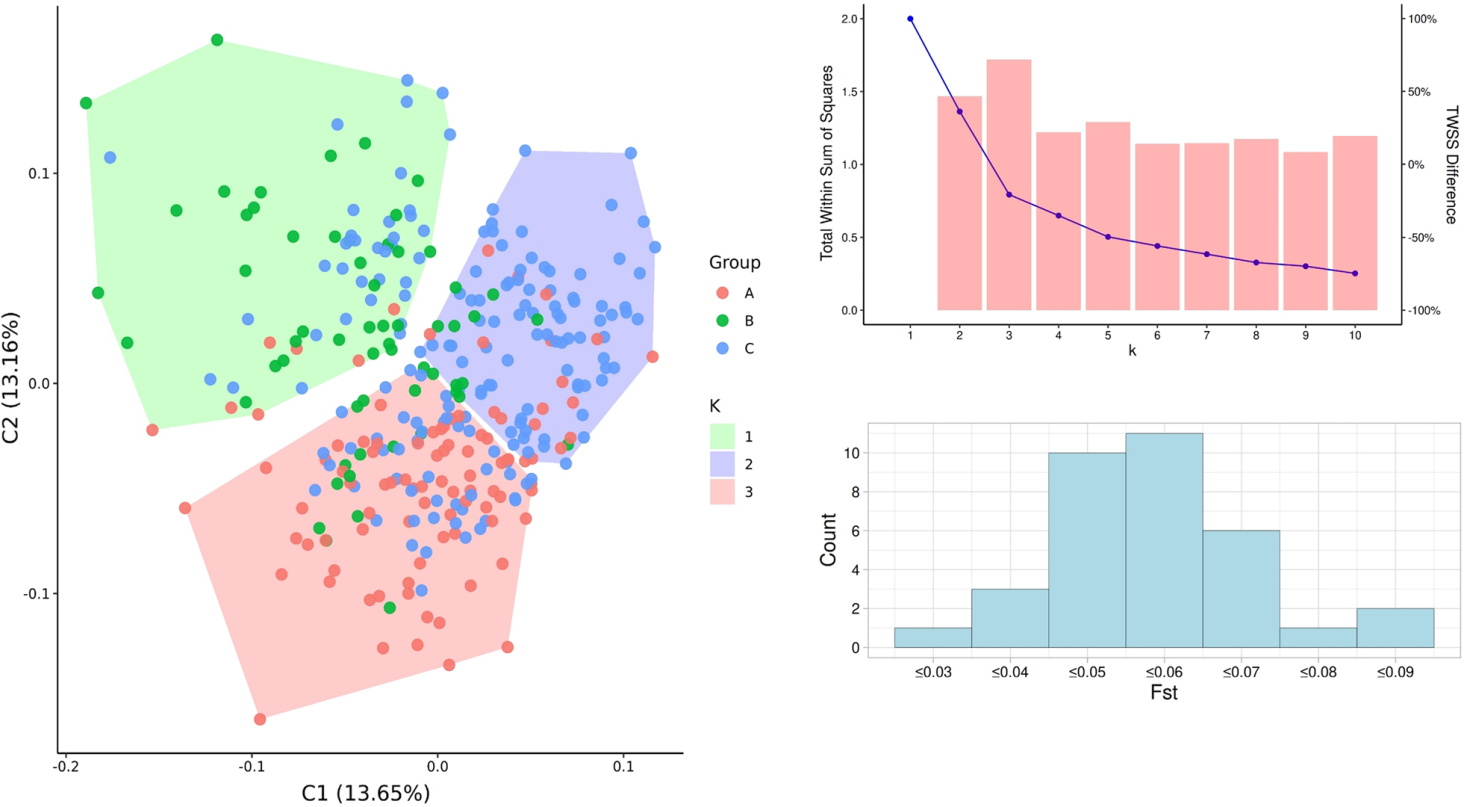
The left image shows the principal component analysis and k-means clustering analysis. The right images show the optimal k for k-means analysis (top right) and SNP F_*ST*_ histogram for the statistically significant microgeographic informative SNPs (bottom right). For the optimal k plot, the blue line with dots represents the TWSS for the k-th value (left axis) and the red bars represent the difference (in percentage) between the TWSS for the k-th value and (k - 1)-th value (right axis). *Abbreviations*: SNP, Single nucleotide; TWSS, total within sum of squares polymorphism

Mean F_*ST*_ values obtained by pairwise comparisons of geographically and behaviorally distinct groups are described in Table 2, considering imputed after pruning (IMPUT) and non-imputed (RAW) data. All geographically distinct populations presented significant F_*ST*_ values, and no behaviorally distinct ones had significant values. For comparison, 39 larvae of *Ny. darlingi* collected from Salvador (Peru), an estimated 465 km from Mâncio Lima (Brazil), were compared with all Brazilian mosquitoes, resulting in significant F_*ST*_ (0.0420; *P*-value < 0.0001) that was 15-fold greater than the highest F_*ST*_ obtained when groups within Mâncio Lima, about 2–3 km apart, were compared. The per SNP permutation analysis resulted in a subset of 34 microgeographic informative SNPs. The results from the PCA and clustering analysis shown in Fig. 3 indicate an optimal k value of k = 3. Clusters 1, 2 and 3 contain 7, 18 and 70 samples from location A, 34, 11 and 15 samples from location B and 35, 83 and 48 samples from location C. The relationship between clusters and locations was found to be not independent (*χ*^2^ = 95.257,* df* = 4, *P*-value < 2.2e-16).

**Table 2:** Mean F_*ST*_ values obtained in pairwise comparisons

Dataset	Group I^a^	Group II^a^	N_M_	Geno	*F*_**ST**_	*p*_VALUE_
IMPUT (imputed after pruning data)	C	B	123,620	188 (84.7)	0.0009	**1.6 × 10**^**-2**^
	C	A	123,620	220(84.6)	0.0012	**1.7 × 10**^**-5**^
	A	B	123,620	130 (84.0)	0.0015	**1.3 × 10**^**-3**^
	Outdoor	Indoor	123,620	170 (84.5)	0.0005	9.4 × 10^-2^
	Dusk	Dawn	123,620	107 (84.5)	0.0005	1.2 × 10^-1^
RAW (non-imputed data)	C	B	15,629	92 (34.5)	0.0008	**3.5 × 10**^**-3**^
	C	A	15,629	86 (33.2)	0.0005	**3.8 × 10**^**-2**^
	A	B	15,629	50 (32.5)	0.0027	**9.8 × 10**^**-4**^
	Outdoor	Indoor	15,629	67(33.5)	0.0001	3.1 × 10^-1^
	Dusk	Dawn	15,629	43(33.5)	0.0001	3.4 × 10^-1^
	C	D	154,813	51 (14.2)	0.0420	**1.0 × 10**^**-4**^

Bold values indicate statistically significant *p* values (*p* < 0.05)

F_*ST*_, Fixation index; *N*_*M*_, number of single nucleotide polymorphisms used; Geno, average of non-missing genotypes per marker (% of total markers)

^a^A, B and C: Locations from Mâncio Lima, Acre at which samples were collected. D: sample location from Loreto, Peru

**Fig. 4:**
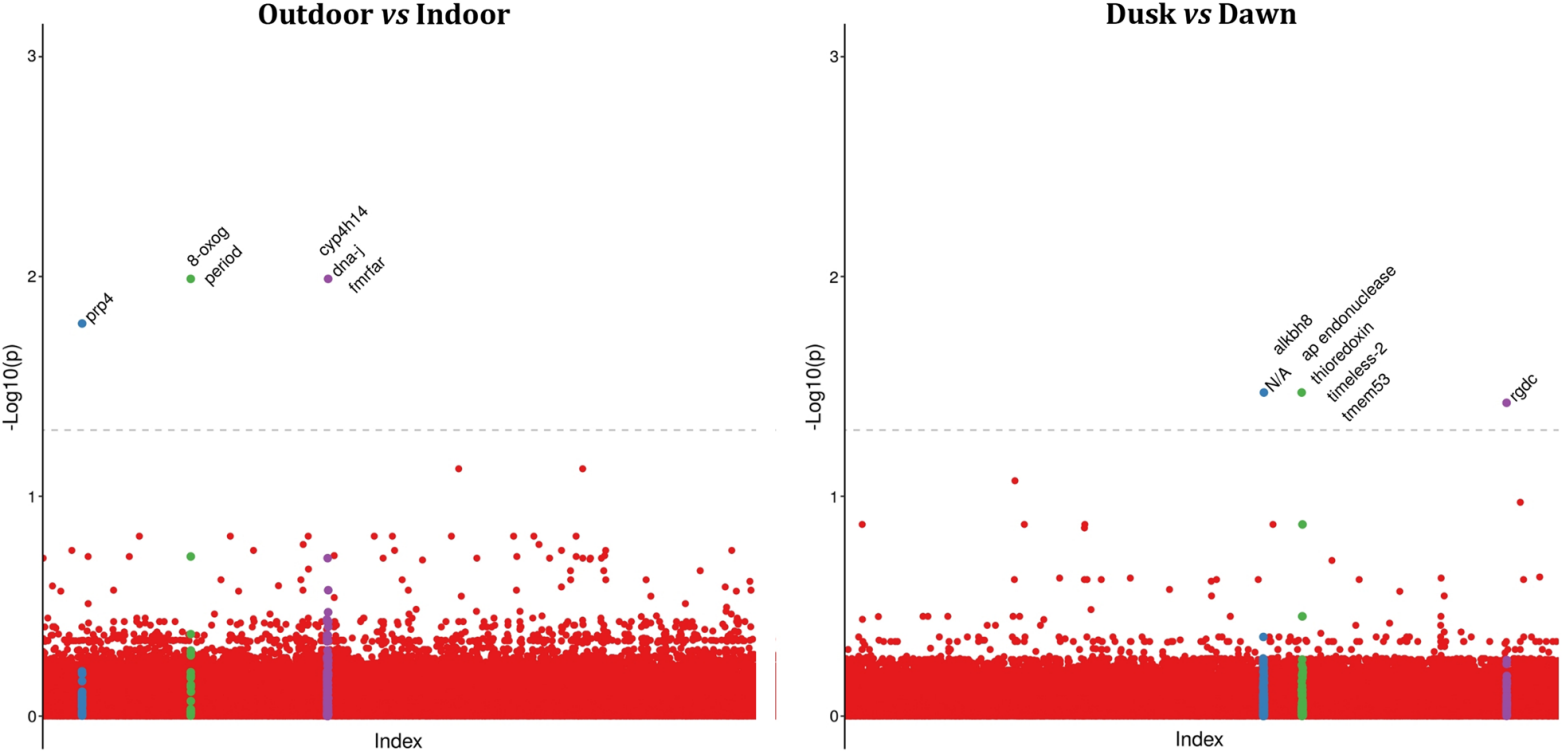
Results for the genome-wide association study on biting behavior (outdoor vs indoor) and blood-seeking (dusk vs dawn). Dashed lines represent false discovery rate-corrected *P*-value threshold of 0.05. Highlighted colors represent the scaffolds containing significantly associated SNPs. Labels represent genes located < 10 kb apart from the significant SNPs

Cochran-Mantel–Haenszel model genome-wide association tests were performed between mosquitoes collected outdoors and indoors (Fg. 4-I; Table 3) and mosquitoes collected during dusk (06:00 to 10:00 PM) and dawn (02:00 to 06:00 AM) (Fig. 4-II; Table 3). For the indoor and outdoor analysis, three different scaffolds had significantly associated SNPs that present genes < 10 kb apart from the significant SNPs (*cyp4H14*, *period* and *prp4*). For the blood-seeking period, three scaffolds had significantly associated SNPs, of which two present genes < 10 kb apart from the significant SNPs (*timeless-2* and *rdgC*) (Fig. 4).

**Table 3:** List of statistically significant markers (*P*_FDR_ < 0.05) and adjacent genes in genome-wide association study for biting behavior and blood-seeking at dusk or dawn

Scaffold	Position	Reference allele	Alternative allele	List of adjacent genes^a^	*P*_FDR_
*Biting behavior*					
ADMH02000641	85279	C	T	FMRFamide receptor [81054:82517]; cytochrome P450 CYP4H14 [87272:89456]; DNA-J [91017:92150]	6.23 × 10^–4^
ADMH02000641	86788	C	A	FMRFamide receptor [81054:82517]; cytochrome P450 CYP4H14 [87272:89456]; DNA-J [91017:92150]	1.03 × 10^–2^
ADMH02001329	14688	T	C	period circadian protein [8167:14819]; 8-oxoguanine DNA glycosylase [6298:7671]	1.03 × 10^–2^
ADMH02002006	31454	G	C	*prp4* [28738:32685]	1.64 × 10^–2^
*Blood-seeking at dusk or dawn*					
ADMH02000323	77474	G	A	–	3.37 × 10^–2^
ADMH02001945	12409	G	A	*timeout/timeless-2* [10017:14540]; transmembrane protein 53-B [15487:16608]; thioredoxin [809:2978]; ap endonuclease [17694:20540]; alkylated DNA repair protein alkB homolog [21608:22713]	3.37 × 10^–2^
ADMH02000929	14323	G	A	retinal degeneration C protein [15781:45295]	3.76 × 10^–2^

*P*_FDR_, False discovery rate-corrected *P*-value (Benjamini–Hochberg procedure)

^a^Adjacent genes located in a maximum range of 10 kb, 5 kb upstream and downstream, are described. Gene start and end positions are represented as follows: [start: end]

## Discussion

### Population stratification and diversity

Significant F_*ST*_ values were observed between groups from different collection sites in both the analyses (imputed and non-imputed data). Considering pairwise comparisons of the groups from Brazil and the comparison between groups from Brazil and Peru, groups collected in Acre showed signs of stratification that were approximately 15-fold lower when compared to the F_*ST*_ values between the groups from Brazil and Loreto, Peru. Although the Mâncio Lima groups showed significance in the permutation tests, the F_*ST*_ values showed a relatively weak signal of stratification. Gélin and collaborators [32] evaluated stratification between populations of *Anopheles gambiae* in Muheza, Tanzania using microsatellite markers from 172 mosquito samples (43, 27 and 102 from Mamboleo, Songa Kibaoni and Zeneth villages, respectively). The linear distances between the studied locations ranged from 5 to 10 km. *F*_ST_ values of 0.001, 0.003 and 0.009 were observed at distances of 6.5, 9.2 and 3.5 km, respectively, but none of the results were significant in the permutation test. Our study presents similar ​​observed values regarding the magnitude of the stratification signal for short distances and, interestingly, the stratification signal of our data is significant. Stratification analysis detected convergent results between the imputed and non-imputed panels, indicating that imputation does not generate significant bias in stratified populations. The groups collected for biting behavior and blood-seeking period did not show significant F_*ST*_ values. The PCA and clustering analysis indicated an optimal k-value of k = 3 because this value had the optimal TWSS difference when compared to k < 3; in addition the TWSS difference reached a plateau when k > 3. The three main clusters indicate a high association with the microgeographic structure, with samples from location A mostly in the third cluster (73.7%), samples from location B mostly in the first cluster (56.7%) and samples from location C mostly in the second cluster (50%). Interestingly, the mosquito population structure presented here seems to be similar with *Plasmodium vivax* population structure reported by Salla and collaborators [33], where the city contains clusters of genetically correlated parasites. We suggest that mosquito population structure could contribute to parasite population structure.

### Genome-wide association study

Our investigation of the gene regions adjacent to the four significant SNPs in the GWAS for biting behavior revealed some genes that should be highlighted, including *prp4* and *CYP4H14* (CYP450 superfamily). Two SNPs (ADMH02000641:85,279 and ADMH02000641:86,788) were < 10 kbp apart from *CYP4H14* (FDR-corrected *P*-value < 0.05). CYP450 is a well-known superfamily containing members that are important in determining insecticide resistance in insects [34], including in anophelines [35–37]. The relationship between the use of pyrethroid insecticides indoors at locations where the samples were collected and the presence of markers associated with CYP450 genes was evident, since individuals with higher degrees of insecticide resistance were found to have a higher chance of survival in an environment where the insecticide was applied. Gao and collaborators [38] studied the response to five different types of insecticides on *Plutella xylostella* based on transcriptome analysis to identify genes that responded to these treatments. The tested insecticides were chlorantraniliprole, cypermethrin, dinotefuran, indoxacarb and spinosad. Differential expression of *prp4* genes was detected, indicating the functional importance of this gene in insecticide resistance. The role of *prp4* is not yet clear, but the results suggest the importance of further studies to disclose the relationship between *prp4* and insecticide resistance.

The results of our GWAS on blood-seeking at dusk or dawn and on adjacent gene regions related to the three significantly associated SNPs highlight two genes and their biological roles. The SNP ADMH02000929:14,323 (FDR-corrected *P*-value < 0.05) is located < 1.5 kb downstream from the retinal degeneration C protein (*rdgC*) gene locus, and the SNP ADMH02001945:12,409 (FDR-corrected *P*-value < 0.05) is located approximately 1 kb downstream from the *timeout/timeless-2* (*tim2* or *timeout*) gene locus. Rhodopsin phosphatase (*rdgC*) plays an important role in the dephosphorylation of rhodopsin Rh1, the most abundant photosensory protein in *Drosophila melanogaster*. Rh1 is required for molecular synchronization with light and circadian rhythm behavior [39]. The loss of the *rdgC* gene function is associated with Rh1 hyperphosphorylation, leading to photoreceptor degeneration in the presence of light in *D. melanogaster* adults [40]. Adewoye[41] described several candidate genes associated with the circadian cycle in *D. melanogaster,* including *rdgC*. *Timeout* is a *timeless* gene (*tim1*) paralog, and both have been described as components of the circadian cycle in *D. melanogaster*. *Timeout* is mainly involved in the perception of luminosity and circadian photoreception in adults [42]. Considering that all of the analyzed samples in the GWAS analysis were adult *Ny. darlingi* (from Brazil), the functional association of* timeout* with the light stimulus at the time that anophelines were collected is rather remarkable. Honnen and collaborators [43] studied differentially expressed genes in response to overnight artificial light treatment in *Culex pipiens* and* timeout* was observed in males.

## Conclusion

The genetic association related to the behavior of females entering houses seems to be selection mediated by the use of indoor insecticides. On the other hand, genetic control of the blood-seeking period could be an ecological adaptation to host availability. Taken together, the data presented here suggest that L-WGS with adequate processing represents a powerful tool to genetically characterize vector populations at a microgeographic scale in malaria transmission areas, as well as for GWAS to disclose behavioral processes, such as the findings that females entering the houses to take a blood meal might be related to a specific *CYP4H14* allele and that female time of blood-seeking is related to circadian rhythm genes.

## Supplementary Information

**Additional file 1: Table A**. List of statistically significant taxonomy identifications using BLAST and* COI* gene sequence as target. Individual sample data can be accessed using the BioSample accession code on NCBI.

## Data Availability

Data are available at NCBI with the following BIOSAMPLE numbers: SAMN17015725 to SAMN17016048 and SAMN21386746 to SAMN21386784 as described in the Additional file 1: Table A.
