# Supplementary material for: Nyssorhynchus darlingi genome-wide studies related to microgeographic dispersion and blood-seeking behavior
Source: Parasit Vectors. 2022 Mar 28;15:106. doi: 10.1186/s13071-022-05219-5 (PMC8961893; doi:10.1186/s13071-022-05219-5)
Supplement: Supplementary file 1 — Additional file 1: Table A. List of statistically significant taxonomy identifications using BLAST and COI gene sequence as target. Individual sample data can be accessed using the BioSample accession code on NCBI. [file 13071_2022_5219_MOESM1_ESM.docx]

# Supplementary Material

| **Table A - List of Statistically significant taxonomy identifications using BLAST and *COI* gene sequence as target. Individual sample data can be accessed using the BioSample accession code on NCBI.** | | | | | |
| --- | --- | --- | --- | --- | --- |
| ID | Taxonomy | E-Value | Seq. Length | Identity (%) | BioSample |
| S00003 | *Nyssorhynchus darlingi* | 0 | 658 | 100.0 | SAMN21386747 |
| S00006 | *Nyssorhynchus darlingi* | 0 | 658 | 100.0 | SAMN21386746 |
| S00007 | *Nyssorhynchus darlingi* | 0 | 658 | 100.0 | SAMN21386748 |
| S00011 | *Nyssorhynchus darlingi* | 0 | 658 | 100.0 | SAMN21386749 |
| S00015 | *Nyssorhynchus darlingi* | 0 | 658 | 100.0 | SAMN21386750 |
| S00018 | *Nyssorhynchus darlingi* | 0 | 658 | 99.8 | SAMN21386751 |
| S00021 | *Nyssorhynchus darlingi* | 0 | 658 | 100.0 | SAMN21386752 |
| S00022 | *Nyssorhynchus darlingi* | 0 | 658 | 100.0 | SAMN21386753 |
| S00023 | *Nyssorhynchus darlingi* | 0 | 658 | 100.0 | SAMN21386754 |
| S00029 | *Nyssorhynchus darlingi* | 0 | 658 | 99.5 | SAMN21386755 |
| S00030 | *Nyssorhynchus darlingi* | 0 | 658 | 100.0 | SAMN21386756 |
| S00032 | *Nyssorhynchus darlingi* | 0 | 658 | 100.0 | SAMN21386757 |
| S00038 | *Nyssorhynchus darlingi* | 0 | 658 | 100.0 | SAMN21386758 |
| S00042 | *Nyssorhynchus darlingi* | 0 | 658 | 100.0 | SAMN21386759 |
| S00043 | *Nyssorhynchus darlingi* | 0 | 658 | 100.0 | SAMN21386760 |
| S00044 | *Nyssorhynchus darlingi* | 0 | 658 | 100.0 | SAMN21386761 |
| S00051 | *Nyssorhynchus darlingi* | 0 | 658 | 100.0 | SAMN21386762 |
| S00052 | *Nyssorhynchus darlingi* | 0 | 658 | 100.0 | SAMN21386763 |
| S00053 | *Nyssorhynchus darlingi* | 0 | 658 | 100.0 | SAMN21386764 |
| S00055 | *Nyssorhynchus darlingi* | 0 | 658 | 100.0 | SAMN21386765 |
| S00057 | *Nyssorhynchus darlingi* | 0 | 658 | 100.0 | SAMN21386766 |
| S00058 | *Nyssorhynchus darlingi* | 0 | 658 | 100.0 | SAMN21386767 |
| S00059 | *Nyssorhynchus darlingi* | 0 | 658 | 100.0 | SAMN21386768 |
| S00061 | *Nyssorhynchus darlingi* | 0 | 658 | 100.0 | SAMN21386769 |
| S00062 | *Nyssorhynchus darlingi* | 0 | 658 | 100.0 | SAMN21386770 |
| S00063 | *Nyssorhynchus darlingi* | 0 | 658 | 99.8 | SAMN21386771 |
| S00064 | *Nyssorhynchus darlingi* | 0 | 658 | 100.0 | SAMN21386772 |
| S00065 | *Nyssorhynchus darlingi* | 0 | 658 | 100.0 | SAMN21386773 |
| S00066 | *Nyssorhynchus darlingi* | 0 | 658 | 100.0 | SAMN21386774 |
| S00067 | *Nyssorhynchus darlingi* | 0 | 658 | 100.0 | SAMN21386775 |
| S00068 | *Nyssorhynchus darlingi* | 0 | 658 | 100.0 | SAMN21386776 |
| S00069 | *Nyssorhynchus darlingi* | 0 | 658 | 100.0 | SAMN21386777 |
| S00070 | *Nyssorhynchus darlingi* | 0 | 658 | 99.8 | SAMN21386778 |
| S00071 | *Nyssorhynchus darlingi* | 0 | 658 | 100.0 | SAMN21386779 |
| S00072 | *Nyssorhynchus darlingi* | 0 | 658 | 99.8 | SAMN21386780 |
| S00073 | *Nyssorhynchus darlingi* | 0 | 658 | 100.0 | SAMN21386781 |
| S00075 | *Nyssorhynchus darlingi* | 0 | 658 | 100.0 | SAMN21386782 |
| S00088 | *Nyssorhynchus darlingi* | 0 | 658 | 100.0 | SAMN21386783 |
| S00092 | *Nyssorhynchus darlingi* | 0 | 658 | 100.0 | SAMN21386784 |
| S00093 | *Nyssorhynchus darlingi* | 0 | 658 | 99.5 | SAMN17015725 |
| S00094 | *Nyssorhynchus darlingi* | 0 | 658 | 99.5 | SAMN17015726 |
| S00095 | *Nyssorhynchus darlingi* | 0 | 658 | 99.5 | SAMN17015727 |
| S00096 | *Nyssorhynchus darlingi* | 0 | 658 | 100.0 | SAMN17015728 |
| S00098 | *Nyssorhynchus darlingi* | 0 | 658 | 100.0 | SAMN17015729 |
| S00099 | *Nyssorhynchus darlingi* | 0 | 658 | 99.5 | SAMN17015730 |
| S00100 | *Nyssorhynchus darlingi* | 0 | 658 | 100.0 | SAMN17015731 |
| S00101 | *Nyssorhynchus darlingi* | 0 | 658 | 100.0 | SAMN17015732 |
| S00102 | *Nyssorhynchus darlingi* | 0 | 658 | 100.0 | SAMN17015733 |
| S00103 | *Nyssorhynchus darlingi* | 0 | 658 | 100.0 | SAMN17015734 |
| S00104 | *Nyssorhynchus darlingi* | 0 | 658 | 99.5 | SAMN17015735 |
| S00106 | *Nyssorhynchus darlingi* | 0 | 658 | 99.5 | SAMN17015736 |
| S00107 | *Nyssorhynchus darlingi* | 0 | 658 | 100.0 | SAMN17015737 |
| S00108 | *Nyssorhynchus darlingi* | 0 | 658 | 100.0 | SAMN17015738 |
| S00111 | *Nyssorhynchus darlingi* | 0 | 658 | 100.0 | SAMN17015739 |
| S00113 | *Nyssorhynchus darlingi* | 0 | 658 | 100.0 | SAMN17015740 |
| S00114 | *Nyssorhynchus darlingi* | 0 | 658 | 100.0 | SAMN17015741 |
| S00115 | *Nyssorhynchus darlingi* | 0 | 658 | 100.0 | SAMN17015742 |
| S00116 | *Nyssorhynchus darlingi* | 0 | 658 | 100.0 | SAMN17015743 |
| S00117 | *Nyssorhynchus darlingi* | 0 | 658 | 100.0 | SAMN17015744 |
| S00118 | *Nyssorhynchus darlingi* | 0 | 658 | 99.8 | SAMN17015745 |
| S00119 | *Nyssorhynchus darlingi* | 0 | 658 | 100.0 | SAMN17015746 |
| S00121 | *Nyssorhynchus darlingi* | 0 | 658 | 99.8 | SAMN17015747 |
| S00122 | *Nyssorhynchus darlingi* | 0 | 658 | 100.0 | SAMN17015748 |
| S00123 | *Nyssorhynchus darlingi* | 0 | 658 | 99.5 | SAMN17015749 |
| S00124 | *Nyssorhynchus darlingi* | 0 | 658 | 100.0 | SAMN17015750 |
| S00125 | *Nyssorhynchus darlingi* | 0 | 658 | 99.5 | SAMN17015751 |
| S00126 | *Nyssorhynchus darlingi* | 0 | 658 | 99.5 | SAMN17015752 |
| S00127 | *Nyssorhynchus darlingi* | 0 | 658 | 99.5 | SAMN17015753 |
| S00128 | *Nyssorhynchus darlingi* | 0 | 658 | 100.0 | SAMN17015754 |
| S00129 | *Nyssorhynchus darlingi* | 0 | 658 | 99.8 | SAMN17015755 |
| S00130 | *Nyssorhynchus darlingi* | 0 | 658 | 100.0 | SAMN17015756 |
| S00131 | *Nyssorhynchus darlingi* | 0 | 658 | 100.0 | SAMN17015757 |
| S00132 | *Nyssorhynchus darlingi* | 0 | 658 | 99.8 | SAMN17015758 |
| S00133 | *Nyssorhynchus darlingi* | 0 | 658 | 99.5 | SAMN17015759 |
| S00134 | *Nyssorhynchus darlingi* | 0 | 658 | 100.0 | SAMN17015760 |
| S00135 | *Nyssorhynchus darlingi* | 0 | 658 | 99.5 | SAMN17015761 |
| S00136 | *Nyssorhynchus darlingi* | 0 | 658 | 99.8 | SAMN17015762 |
| S00137 | *Nyssorhynchus darlingi* | 0 | 658 | 99.8 | SAMN17015763 |
| S00139 | *Nyssorhynchus darlingi* | 0 | 658 | 100.0 | SAMN17015764 |
| S00140 | *Nyssorhynchus darlingi* | 0 | 658 | 100.0 | SAMN17015765 |
| S00141 | *Nyssorhynchus darlingi* | 0 | 658 | 100.0 | SAMN17015766 |
| S00142 | *Nyssorhynchus darlingi* | 0 | 658 | 100.0 | SAMN17015767 |
| S00143 | *Nyssorhynchus darlingi* | 0 | 658 | 100.0 | SAMN17015768 |
| S00144 | *Nyssorhynchus darlingi* | 0 | 658 | 100.0 | SAMN17015769 |
| S00145 | *Nyssorhynchus darlingi* | 0 | 658 | 100.0 | SAMN17015770 |
| S00146 | *Nyssorhynchus darlingi* | 0 | 658 | 99.5 | SAMN17015771 |
| S00147 | *Nyssorhynchus darlingi* | 0 | 658 | 100.0 | SAMN17015772 |
| S00149 | *Nyssorhynchus darlingi* | 0 | 658 | 99.8 | SAMN17015773 |
| S00150 | *Nyssorhynchus darlingi* | 0 | 658 | 100.0 | SAMN17015774 |
| S00151 | *Nyssorhynchus darlingi* | 0 | 658 | 100.0 | SAMN17015775 |
| S00152 | *Nyssorhynchus darlingi* | 0 | 658 | 100.0 | SAMN17015776 |
| S00153 | *Nyssorhynchus darlingi* | 0 | 658 | 100.0 | SAMN17015777 |
| S00154 | *Nyssorhynchus darlingi* | 0 | 658 | 100.0 | SAMN17015778 |
| S00155 | *Nyssorhynchus darlingi* | 0 | 658 | 100.0 | SAMN17015779 |
| S00156 | *Nyssorhynchus darlingi* | 0 | 658 | 100.0 | SAMN17015780 |
| S00157 | *Nyssorhynchus darlingi* | 0 | 658 | 100.0 | SAMN17015781 |
| S00158 | *Nyssorhynchus darlingi* | 0 | 658 | 100.0 | SAMN17015782 |
| S00169 | *Nyssorhynchus darlingi* | 0 | 658 | 100.0 | SAMN17015783 |
| S00170 | *Nyssorhynchus darlingi* | 0 | 658 | 100.0 | SAMN17015784 |
| S00171 | *Nyssorhynchus darlingi* | 0 | 658 | 100.0 | SAMN17015785 |
| S00172 | *Nyssorhynchus darlingi* | 0 | 658 | 99.5 | SAMN17015786 |
| S00173 | *Nyssorhynchus darlingi* | 0 | 658 | 100.0 | SAMN17015787 |
| S00174 | *Nyssorhynchus darlingi* | 0 | 658 | 99.5 | SAMN17015788 |
| S00175 | *Nyssorhynchus darlingi* | 0 | 658 | 100.0 | SAMN17015789 |
| S00176 | *Nyssorhynchus darlingi* | 0 | 658 | 98.9 | SAMN17015790 |
| S00181 | *Nyssorhynchus darlingi* | 0 | 658 | 99.8 | SAMN17015791 |
| S00182 | *Nyssorhynchus darlingi* | 0 | 658 | 100.0 | SAMN17015792 |
| S00183 | *Nyssorhynchus darlingi* | 0 | 658 | 99.5 | SAMN17015793 |
| S00184 | *Nyssorhynchus darlingi* | 0 | 658 | 100.0 | SAMN17015794 |
| S00185 | *Nyssorhynchus darlingi* | 0 | 658 | 99.5 | SAMN17015795 |
| S00186 | *Nyssorhynchus darlingi* | 0 | 658 | 100.0 | SAMN17015796 |
| S00187 | *Nyssorhynchus darlingi* | 0 | 658 | 100.0 | SAMN17015797 |
| S00188 | *Nyssorhynchus darlingi* | 0 | 658 | 100.0 | SAMN17015798 |
| S00189 | *Nyssorhynchus darlingi* | 0 | 658 | 100.0 | SAMN17015799 |
| S00190 | *Nyssorhynchus darlingi* | 0 | 658 | 99.5 | SAMN17015800 |
| S00191 | *Nyssorhynchus darlingi* | 0 | 658 | 100.0 | SAMN17015801 |
| S00192 | *Nyssorhynchus darlingi* | 0 | 658 | 99.8 | SAMN17015802 |
| S00193 | *Nyssorhynchus darlingi* | 0 | 658 | 99.5 | SAMN17015803 |
| S00194 | *Nyssorhynchus darlingi* | 0 | 658 | 99.8 | SAMN17015804 |
| S00200 | *Nyssorhynchus darlingi* | 0 | 658 | 100.0 | SAMN17015806 |
| S00201 | *Nyssorhynchus darlingi* | 0 | 658 | 100.0 | SAMN17015807 |
| S00202 | *Nyssorhynchus darlingi* | 0 | 658 | 99.5 | SAMN17015808 |
| S00203 | *Nyssorhynchus darlingi* | 0 | 658 | 100.0 | SAMN17015809 |
| S00204 | *Nyssorhynchus darlingi* | 0 | 658 | 100.0 | SAMN17015810 |
| S00205 | *Nyssorhynchus darlingi* | 0 | 658 | 100.0 | SAMN17015811 |
| S00206 | *Nyssorhynchus darlingi* | 0 | 658 | 99.5 | SAMN17015812 |
| S00207 | *Nyssorhynchus darlingi* | 0 | 658 | 99.5 | SAMN17015813 |
| S00208 | *Nyssorhynchus darlingi* | 0 | 658 | 99.5 | SAMN17015814 |
| S00210 | *Nyssorhynchus darlingi* | 0 | 658 | 100.0 | SAMN17015815 |
| S00211 | *Nyssorhynchus darlingi* | 0 | 658 | 100.0 | SAMN17015816 |
| S00212 | *Nyssorhynchus darlingi* | 0 | 646 | 95.7 | SAMN17015817 |
| S00213 | *Nyssorhynchus darlingi* | 0 | 658 | 99.7 | SAMN17015818 |
| S00214 | *Nyssorhynchus darlingi* | 0 | 652 | 99.5 | SAMN17015819 |
| S00219 | *Nyssorhynchus darlingi* | 0 | 658 | 100.0 | SAMN17015820 |
| S00221 | *Nyssorhynchus darlingi* | 0 | 658 | 99.5 | SAMN17015821 |
| S00222 | *Nyssorhynchus darlingi* | 0 | 658 | 99.8 | SAMN17015822 |
| S00224 | *Nyssorhynchus darlingi* | 0 | 658 | 100.0 | SAMN17015823 |
| S00226 | *Nyssorhynchus darlingi* | 0 | 478 | 100.0 | SAMN17015824 |
| S00227 | *Nyssorhynchus darlingi* | 0 | 658 | 100.0 | SAMN17015825 |
| S00228 | *Nyssorhynchus darlingi* | 0 | 658 | 99.5 | SAMN17015826 |
| S00229 | *Nyssorhynchus darlingi* | 0 | 658 | 100.0 | SAMN17015827 |
| S00230 | *Nyssorhynchus darlingi* | 0 | 658 | 99.5 | SAMN17015828 |
| S00231 | *Nyssorhynchus darlingi* | 0 | 658 | 99.5 | SAMN17015829 |
| S00232 | *Nyssorhynchus darlingi* | 0 | 658 | 100.0 | SAMN17015830 |
| S00233 | *Nyssorhynchus darlingi* | 0 | 658 | 98.6 | SAMN17015831 |
| S00234 | *Nyssorhynchus darlingi* | 0 | 658 | 99.5 | SAMN17015832 |
| S00235 | *Nyssorhynchus darlingi* | 0 | 658 | 100.0 | SAMN17015833 |
| S00236 | *Nyssorhynchus darlingi* | 0 | 653 | 99.5 | SAMN17015834 |
| S00237 | *Nyssorhynchus darlingi* | 0 | 658 | 100.0 | SAMN17015835 |
| S00238 | *Nyssorhynchus darlingi* | 0 | 658 | 99.7 | SAMN17015836 |
| S00239 | *Nyssorhynchus darlingi* | 0 | 658 | 100.0 | SAMN17015837 |
| S00242 | *Nyssorhynchus darlingi* | 0 | 652 | 96.9 | SAMN17015838 |
| S00244 | *Nyssorhynchus darlingi* | 0 | 658 | 99.5 | SAMN17015839 |
| S00245 | *Nyssorhynchus darlingi* | 0 | 658 | 100.0 | SAMN17015840 |
| S00246 | *Nyssorhynchus darlingi* | 0 | 658 | 100.0 | SAMN17015841 |
| S00247 | *Nyssorhynchus darlingi* | 0 | 658 | 100.0 | SAMN17015842 |
| S00248 | *Nyssorhynchus darlingi* | 0 | 658 | 100.0 | SAMN17015843 |
| S00249 | *Nyssorhynchus darlingi* | 0 | 658 | 100.0 | SAMN17015844 |
| S00250 | *Nyssorhynchus darlingi* | 0 | 658 | 100.0 | SAMN17015845 |
| S00251 | *Nyssorhynchus darlingi* | 0 | 658 | 99.2 | SAMN17015846 |
| S00252 | *Nyssorhynchus darlingi* | 0 | 658 | 99.5 | SAMN17015847 |
| S00254 | *Nyssorhynchus darlingi* | 0 | 490 | 100.0 | SAMN17015848 |
| S00255 | *Nyssorhynchus darlingi* | 0 | 658 | 100.0 | SAMN17015849 |
| S00256 | *Nyssorhynchus darlingi* | 0 | 658 | 99.8 | SAMN17015850 |
| S00258 | *Nyssorhynchus darlingi* | 0 | 658 | 100.0 | SAMN17015851 |
| S00259 | *Nyssorhynchus darlingi* | 0 | 658 | 100.0 | SAMN17015852 |
| S00260 | *Nyssorhynchus darlingi* | 0 | 656 | 99.4 | SAMN17015853 |
| S00262 | *Nyssorhynchus darlingi* | 0 | 658 | 98.5 | SAMN17015854 |
| S00270 | *Nyssorhynchus darlingi* | 0 | 658 | 100.0 | SAMN17015855 |
| S00271 | *Nyssorhynchus darlingi* | 0 | 658 | 99.5 | SAMN17015856 |
| S00272 | *Nyssorhynchus darlingi* | 0 | 486 | 99.8 | SAMN17015857 |
| S00274 | *Nyssorhynchus darlingi* | 0 | 519 | 97.1 | SAMN17015859 |
| S00275 | *Nyssorhynchus darlingi* | 0 | 500 | 100.0 | SAMN17015860 |
| S00284 | *Nyssorhynchus darlingi* | 0 | 658 | 100.0 | SAMN17015862 |
| S00287 | *Nyssorhynchus darlingi* | 0 | 658 | 100.0 | SAMN17015863 |
| S00288 | *Nyssorhynchus darlingi* | 0 | 658 | 99.5 | SAMN17015864 |
| S00295 | *Nyssorhynchus darlingi* | 0 | 501 | 100.0 | SAMN17015866 |
| S00297 | *Nyssorhynchus darlingi* | 0 | 658 | 99.7 | SAMN17015868 |
| S00298 | *Nyssorhynchus darlingi* | 0 | 658 | 97.3 | SAMN17015869 |
| S00299 | *Nyssorhynchus darlingi* | 0 | 483 | 94.4 | SAMN17015870 |
| S00301 | *Nyssorhynchus darlingi* | 0 | 658 | 100.0 | SAMN17015871 |
| S00302 | *Nyssorhynchus darlingi* | 0 | 658 | 100.0 | SAMN17015872 |
| S00303 | *Nyssorhynchus darlingi* | 0 | 658 | 100.0 | SAMN17015873 |
| S00304 | *Nyssorhynchus darlingi* | 0 | 658 | 99.5 | SAMN17015874 |
| S00305 | *Nyssorhynchus darlingi* | 0 | 658 | 99.5 | SAMN17015875 |
| S00306 | *Nyssorhynchus darlingi* | 0 | 658 | 100.0 | SAMN17015876 |
| S00307 | *Nyssorhynchus darlingi* | 0 | 658 | 99.8 | SAMN17015877 |
| S00308 | *Nyssorhynchus darlingi* | 0 | 658 | 99.5 | SAMN17015878 |
| S00309 | *Nyssorhynchus darlingi* | 0 | 658 | 100.0 | SAMN17015879 |
| S00310 | *Nyssorhynchus darlingi* | 0 | 658 | 100.0 | SAMN17015880 |
| S00311 | *Nyssorhynchus darlingi* | 0 | 658 | 99.5 | SAMN17015881 |
| S00312 | *Nyssorhynchus darlingi* | 0 | 658 | 100.0 | SAMN17015882 |
| S00313 | *Nyssorhynchus darlingi* | 0 | 658 | 99.5 | SAMN17015883 |
| S00314 | *Nyssorhynchus darlingi* | 0 | 658 | 100.0 | SAMN17015884 |
| S00315 | *Nyssorhynchus darlingi* | 0 | 658 | 99.8 | SAMN17015885 |
| S00316 | *Nyssorhynchus darlingi* | 0 | 658 | 99.5 | SAMN17015886 |
| S00317 | *Nyssorhynchus darlingi* | 0 | 658 | 100.0 | SAMN17015887 |
| S00318 | *Nyssorhynchus darlingi* | 0 | 658 | 99.5 | SAMN17015888 |
| S00319 | *Nyssorhynchus darlingi* | 0 | 658 | 100.0 | SAMN17015889 |
| S00320 | *Nyssorhynchus darlingi* | 0 | 658 | 100.0 | SAMN17015890 |
| S00321 | *Nyssorhynchus darlingi* | 0 | 658 | 99.5 | SAMN17015891 |
| S00322 | *Nyssorhynchus darlingi* | 0 | 658 | 100.0 | SAMN17015892 |
| S00323 | *Nyssorhynchus darlingi* | 0 | 658 | 100.0 | SAMN17015893 |
| S00324 | *Nyssorhynchus darlingi* | 0 | 658 | 99.4 | SAMN17015894 |
| S00325 | *Nyssorhynchus darlingi* | 0 | 658 | 100.0 | SAMN17015895 |
| S00326 | *Nyssorhynchus darlingi* | 0 | 658 | 100.0 | SAMN17015896 |
| S00327 | *Nyssorhynchus darlingi* | 0 | 658 | 99.4 | SAMN17015897 |
| S00328 | *Nyssorhynchus darlingi* | 0 | 658 | 100.0 | SAMN17015898 |
| S00329 | *Nyssorhynchus darlingi* | 0 | 658 | 99.8 | SAMN17015899 |
| S00332 | *Nyssorhynchus darlingi* | 0 | 658 | 99.5 | SAMN17015900 |
| S00334 | *Nyssorhynchus darlingi* | 0 | 658 | 100.0 | SAMN17015901 |
| S00335 | *Nyssorhynchus darlingi* | 0 | 658 | 100.0 | SAMN17015902 |
| S00336 | *Nyssorhynchus darlingi* | 0 | 658 | 99.5 | SAMN17015903 |
| S00337 | *Nyssorhynchus darlingi* | 0 | 658 | 99.5 | SAMN17015904 |
| S00342 | *Nyssorhynchus darlingi* | 0 | 658 | 99.5 | SAMN17015905 |
| S00345 | *Nyssorhynchus darlingi* | 0 | 658 | 100.0 | SAMN17015906 |
| S00347 | *Nyssorhynchus darlingi* | 0 | 658 | 100.0 | SAMN17015907 |
| S00348 | *Nyssorhynchus darlingi* | 0 | 658 | 100.0 | SAMN17015908 |
| S00349 | *Nyssorhynchus darlingi* | 0 | 658 | 99.8 | SAMN17015909 |
| S00351 | *Nyssorhynchus darlingi* | 0 | 658 | 100.0 | SAMN17015910 |
| S00352 | *Nyssorhynchus darlingi* | 0 | 658 | 100.0 | SAMN17015911 |
| S00353 | *Nyssorhynchus darlingi* | 0 | 658 | 99.8 | SAMN17015912 |
| S00354 | *Nyssorhynchus darlingi* | 0 | 658 | 100.0 | SAMN17015913 |
| S00355 | *Nyssorhynchus darlingi* | 0 | 658 | 100.0 | SAMN17015914 |
| S00356 | *Nyssorhynchus darlingi* | 0 | 658 | 100.0 | SAMN17015915 |
| S00357 | *Nyssorhynchus darlingi* | 0 | 658 | 100.0 | SAMN17015916 |
| S00358 | *Nyssorhynchus darlingi* | 0 | 658 | 100.0 | SAMN17015917 |
| S00359 | *Nyssorhynchus darlingi* | 0 | 658 | 100.0 | SAMN17015918 |
| S00360 | *Nyssorhynchus darlingi* | 0 | 658 | 100.0 | SAMN17015919 |
| S00361 | *Nyssorhynchus darlingi* | 0 | 658 | 99.8 | SAMN17015920 |
| S00362 | *Nyssorhynchus darlingi* | 0 | 658 | 100.0 | SAMN17015921 |
| S00363 | *Nyssorhynchus darlingi* | 0 | 658 | 100.0 | SAMN17015922 |
| S00364 | *Nyssorhynchus darlingi* | 0 | 658 | 100.0 | SAMN17015923 |
| S00365 | *Nyssorhynchus darlingi* | 0 | 658 | 99.4 | SAMN17015924 |
| S00366 | *Nyssorhynchus darlingi* | 0 | 658 | 99.7 | SAMN17015925 |
| S00367 | *Nyssorhynchus darlingi* | 0 | 658 | 99.5 | SAMN17015926 |
| S00368 | *Nyssorhynchus darlingi* | 0 | 658 | 100.0 | SAMN17015927 |
| S00369 | *Nyssorhynchus darlingi* | 0 | 658 | 100.0 | SAMN17015928 |
| S00370 | *Nyssorhynchus darlingi* | 0 | 658 | 99.5 | SAMN17015929 |
| S00371 | *Nyssorhynchus darlingi* | 0 | 658 | 99.5 | SAMN17015930 |
| S00372 | *Nyssorhynchus darlingi* | 0 | 658 | 100.0 | SAMN17015931 |
| S00373 | *Nyssorhynchus darlingi* | 0 | 658 | 100.0 | SAMN17015932 |
| S00374 | *Nyssorhynchus darlingi* | 0 | 658 | 100.0 | SAMN17015933 |
| S00375 | *Nyssorhynchus darlingi* | 0 | 658 | 100.0 | SAMN17015934 |
| S00377 | *Nyssorhynchus darlingi* | 0 | 658 | 99.8 | SAMN17015935 |
| S00378 | *Nyssorhynchus darlingi* | 0 | 658 | 100.0 | SAMN17015936 |
| S00379 | *Nyssorhynchus darlingi* | 0 | 658 | 100.0 | SAMN17015937 |
| S00380 | *Nyssorhynchus darlingi* | 0 | 658 | 100.0 | SAMN17015938 |
| S00382 | *Nyssorhynchus darlingi* | 0 | 658 | 99.8 | SAMN17015939 |
| S00383 | *Nyssorhynchus darlingi* | 0 | 658 | 99.5 | SAMN17015940 |
| S00384 | *Nyssorhynchus darlingi* | 0 | 658 | 100.0 | SAMN17015941 |
| S00385 | *Nyssorhynchus darlingi* | 0 | 658 | 99.8 | SAMN17015942 |
| S00386 | *Nyssorhynchus darlingi* | 0 | 658 | 100.0 | SAMN17015943 |
| S00387 | *Nyssorhynchus darlingi* | 0 | 658 | 100.0 | SAMN17015944 |
| S00389 | *Nyssorhynchus darlingi* | 0 | 658 | 99.5 | SAMN17015945 |
| S00390 | *Nyssorhynchus darlingi* | 0 | 658 | 100.0 | SAMN17015946 |
| S00391 | *Nyssorhynchus darlingi* | 0 | 658 | 100.0 | SAMN17015947 |
| S00393 | *Nyssorhynchus darlingi* | 0 | 658 | 100.0 | SAMN17015948 |
| S00394 | *Nyssorhynchus darlingi* | 0 | 658 | 99.8 | SAMN17015949 |
| S00395 | *Nyssorhynchus darlingi* | 0 | 658 | 100.0 | SAMN17015950 |
| S00396 | *Nyssorhynchus darlingi* | 0 | 658 | 100.0 | SAMN17015951 |
| S00397 | *Nyssorhynchus darlingi* | 0 | 658 | 99.5 | SAMN17015952 |
| S00398 | *Nyssorhynchus darlingi* | 0 | 658 | 100.0 | SAMN17015953 |
| S00399 | *Nyssorhynchus darlingi* | 0 | 658 | 100.0 | SAMN17015954 |
| S00400 | *Nyssorhynchus darlingi* | 0 | 658 | 100.0 | SAMN17015955 |
| S00401 | *Nyssorhynchus darlingi* | 0 | 658 | 100.0 | SAMN17015956 |
| S00402 | *Nyssorhynchus darlingi* | 0 | 658 | 100.0 | SAMN17015957 |
| S00403 | *Nyssorhynchus darlingi* | 0 | 658 | 99.5 | SAMN17015958 |
| S00404 | *Nyssorhynchus darlingi* | 0 | 658 | 99.5 | SAMN17015959 |
| S00405 | *Nyssorhynchus darlingi* | 0 | 658 | 99.5 | SAMN17015960 |
| S00406 | *Nyssorhynchus darlingi* | 0 | 658 | 99.5 | SAMN17015961 |
| S00407 | *Nyssorhynchus darlingi* | 0 | 658 | 99.5 | SAMN17015962 |
| S00409 | *Nyssorhynchus darlingi* | 0 | 658 | 99.5 | SAMN17015963 |
| S00411 | *Nyssorhynchus darlingi* | 0 | 658 | 99.5 | SAMN17015964 |
| S00412 | *Nyssorhynchus darlingi* | 0 | 658 | 100.0 | SAMN17015965 |
| S00413 | *Nyssorhynchus darlingi* | 0 | 658 | 99.5 | SAMN17015966 |
| S00414 | *Nyssorhynchus darlingi* | 0 | 658 | 100.0 | SAMN17015967 |
| S00415 | *Nyssorhynchus darlingi* | 0 | 658 | 100.0 | SAMN17015968 |
| S00416 | *Nyssorhynchus darlingi* | 0 | 658 | 100.0 | SAMN17015969 |
| S00417 | *Nyssorhynchus darlingi* | 0 | 658 | 100.0 | SAMN17015970 |
| S00418 | *Nyssorhynchus darlingi* | 0 | 658 | 99.5 | SAMN17015971 |
| S00421 | *Nyssorhynchus darlingi* | 0 | 658 | 100.0 | SAMN17015973 |
| S00423 | *Nyssorhynchus darlingi* | 0 | 658 | 99.5 | SAMN17015974 |
| S00425 | *Nyssorhynchus darlingi* | 0 | 658 | 99.5 | SAMN17015975 |
| S00426 | *Nyssorhynchus darlingi* | 0 | 658 | 100.0 | SAMN17015976 |
| S00427 | *Nyssorhynchus darlingi* | 0 | 658 | 100.0 | SAMN17015977 |
| S00428 | *Nyssorhynchus darlingi* | 0 | 658 | 100.0 | SAMN17015978 |
| S00429 | *Nyssorhynchus darlingi* | 0 | 658 | 99.4 | SAMN17015979 |
| S00430 | *Nyssorhynchus darlingi* | 0 | 658 | 100.0 | SAMN17015980 |
| S00431 | *Nyssorhynchus darlingi* | 0 | 658 | 100.0 | SAMN17015981 |
| S00432 | *Nyssorhynchus darlingi* | 0 | 658 | 100.0 | SAMN17015982 |
| S00433 | *Nyssorhynchus darlingi* | 0 | 658 | 99.1 | SAMN17015983 |
| S00434 | *Nyssorhynchus darlingi* | 0 | 658 | 100.0 | SAMN17015984 |
| S00435 | *Nyssorhynchus darlingi* | 0 | 658 | 100.0 | SAMN17015985 |
| S00436 | *Nyssorhynchus darlingi* | 0 | 658 | 100.0 | SAMN17015986 |
| S00437 | *Nyssorhynchus darlingi* | 0 | 658 | 100.0 | SAMN17015987 |
| S00438 | *Nyssorhynchus darlingi* | 0 | 658 | 100.0 | SAMN17015988 |
| S00439 | *Nyssorhynchus darlingi* | 0 | 658 | 100.0 | SAMN17015989 |
| S00440 | *Nyssorhynchus darlingi* | 0 | 658 | 100.0 | SAMN17015990 |
| S00441 | *Nyssorhynchus darlingi* | 0 | 658 | 98.8 | SAMN17015991 |
| S00442 | *Nyssorhynchus darlingi* | 0 | 556 | 97.8 | SAMN17015992 |
| S00443 | *Nyssorhynchus darlingi* | 0 | 658 | 97.7 | SAMN17015993 |
| S00444 | *Nyssorhynchus darlingi* | 0 | 658 | 98.6 | SAMN17015994 |
| S00446 | *Nyssorhynchus darlingi* | 0 | 658 | 97.6 | SAMN17015995 |
| S00447 | *Nyssorhynchus darlingi* | 0 | 657 | 98.2 | SAMN17015996 |
| S00448 | *Nyssorhynchus darlingi* | 0 | 658 | 98.2 | SAMN17015997 |
| S00449 | *Nyssorhynchus darlingi* | 0 | 658 | 98.5 | SAMN17015998 |
| S00452 | *Nyssorhynchus darlingi* | 0 | 658 | 98.6 | SAMN17016000 |
| S00453 | *Nyssorhynchus darlingi* | 0 | 658 | 98.3 | SAMN17016001 |
| S00454 | *Nyssorhynchus darlingi* | 0 | 385 | 98.7 | SAMN17016002 |
| S00455 | *Nyssorhynchus darlingi* | 0 | 658 | 99.7 | SAMN17016003 |
| S00457 | *Nyssorhynchus darlingi* | 0 | 658 | 97.6 | SAMN17016004 |
| S00458 | *Nyssorhynchus darlingi* | 0 | 658 | 97.9 | SAMN17016005 |
| S00465 | *Nyssorhynchus darlingi* | 0 | 658 | 99.5 | SAMN17016007 |
| S00466 | *Nyssorhynchus darlingi* | 0 | 658 | 97.6 | SAMN17016008 |
| S00468 | *Nyssorhynchus darlingi* | 0 | 658 | 98.3 | SAMN17016009 |
| S00469 | *Nyssorhynchus darlingi* | 0 | 658 | 98.0 | SAMN17016010 |
| S00470 | *Nyssorhynchus darlingi* | 0 | 658 | 99.5 | SAMN17016011 |
| S00471 | *Nyssorhynchus darlingi* | 0 | 658 | 99.5 | SAMN17016012 |
| S00472 | *Nyssorhynchus darlingi* | 0 | 658 | 99.5 | SAMN17016013 |
| S00473 | *Nyssorhynchus darlingi* | 0 | 658 | 98.2 | SAMN17016014 |
| S00474 | *Nyssorhynchus darlingi* | 0 | 658 | 98.3 | SAMN17016015 |
| S00475 | *Nyssorhynchus darlingi* | 0 | 658 | 99.1 | SAMN17016016 |
| S00476 | *Nyssorhynchus darlingi* | 0 | 658 | 98.5 | SAMN17016017 |
| S00481 | *Nyssorhynchus darlingi* | 0 | 658 | 98.2 | SAMN17016018 |
| S00482 | *Nyssorhynchus darlingi* | 0 | 658 | 98.2 | SAMN17016019 |
| S00483 | *Nyssorhynchus darlingi* | 0 | 658 | 97.1 | SAMN17016020 |
| S00484 | *Nyssorhynchus darlingi* | 0 | 658 | 97.9 | SAMN17016021 |
| S00485 | *Nyssorhynchus darlingi* | 0 | 658 | 98.3 | SAMN17016022 |
| S00488 | *Nyssorhynchus darlingi* | 0 | 658 | 99.5 | SAMN17016023 |
| S00489 | *Nyssorhynchus darlingi* | 0 | 658 | 100.0 | SAMN17016024 |
| S00490 | *Nyssorhynchus darlingi* | 0 | 658 | 100.0 | SAMN17016025 |
| S00491 | *Nyssorhynchus darlingi* | 0 | 658 | 100.0 | SAMN17016026 |
| S00493 | *Nyssorhynchus darlingi* | 0 | 658 | 99.5 | SAMN17016027 |
| S00499 | *Nyssorhynchus darlingi* | 0 | 658 | 100.0 | SAMN17016028 |
| S00500 | *Nyssorhynchus darlingi* | 0 | 658 | 99.5 | SAMN17016029 |
| S00501 | *Nyssorhynchus darlingi* | 0 | 658 | 100.0 | SAMN17016030 |
| S00502 | *Nyssorhynchus darlingi* | 0 | 658 | 99.5 | SAMN17016031 |
| S00503 | *Nyssorhynchus darlingi* | 0 | 658 | 100.0 | SAMN17016032 |
| S00504 | *Nyssorhynchus darlingi* | 0 | 658 | 99.5 | SAMN17016033 |
| S00505 | *Nyssorhynchus darlingi* | 0 | 658 | 99.5 | SAMN17016034 |
| S00506 | *Nyssorhynchus darlingi* | 0 | 658 | 99.5 | SAMN17016035 |
| S00508 | *Nyssorhynchus darlingi* | 0 | 658 | 99.5 | SAMN17016036 |
| S00509 | *Nyssorhynchus darlingi* | 0 | 658 | 99.5 | SAMN17016037 |
| S00511 | *Nyssorhynchus darlingi* | 0 | 658 | 100.0 | SAMN17016038 |
| S00512 | *Nyssorhynchus darlingi* | 0 | 658 | 99.8 | SAMN17016039 |
| S00513 | *Nyssorhynchus darlingi* | 0 | 658 | 99.5 | SAMN17016040 |
| S00514 | *Nyssorhynchus darlingi* | 0 | 658 | 99.5 | SAMN17016041 |
| S00518 | *Nyssorhynchus darlingi* | 0 | 658 | 99.8 | SAMN17016042 |
| S00519 | *Nyssorhynchus darlingi* | 0 | 658 | 99.5 | SAMN17016043 |
| S00520 | *Nyssorhynchus darlingi* | 0 | 658 | 100.0 | SAMN17016044 |
| S00521 | *Nyssorhynchus darlingi* | 0 | 658 | 100.0 | SAMN17016045 |
| S00522 | *Nyssorhynchus darlingi* | 0 | 658 | 100.0 | SAMN17016046 |
| S00523 | *Nyssorhynchus darlingi* | 0 | 658 | 100.0 | SAMN17016047 |
| S00524 | *Nyssorhynchus darlingi* | 0 | 658 | 100.0 | SAMN17016048 |
| S00281 | *Nyssorhynchus darlingi* | 9.4E-175 | 333 | 100.0 | SAMN17015861 |
| S00459 | *Nyssorhynchus darlingi* | 3.4E-169 | 357 | 96.4 | SAMN17016006 |
| S00273 | *Nyssorhynchus darlingi* | 1.6E-157 | 302 | 100.0 | SAMN17015858 |
| S00450 | *Nyssorhynchus darlingi* | 2.7E-150 | 337 | 92.9 | SAMN17015999 |
| S00296 | *Nyssorhynchus darlingi* | 1.0E-99 | 204 | 98.5 | SAMN17015867 |
| ID: Individual identification code used in this study. Taxonomy: Taxonomy report from blastn results. | | | | | |
